# The lack of a big picture in tuberculosis: the clinical point of view, the problems of experimental modeling and immunomodulation. The factors we should consider when designing novel treatment strategies

**DOI:** 10.3389/fmicb.2014.00055

**Published:** 2014-02-14

**Authors:** Cristina Vilaplana, Pere-Joan Cardona

**Affiliations:** Unitat de Tuberculosi Experimental, Fundació Institut Germans Trias i Pujol, Universitat Autònoma de Barcelona, CIBER Enfermedades RespiratoriasBadalona, Spain

**Keywords:** tuberculosis, tuberculosis disease, inflammation, liquefaction, experimental models, Kramnik model, *M. tuberculosis*

## Abstract

This short review explores the large gap between clinical issues and basic science, and suggests why tuberculosis research should focus on redirect the immune system and not only on eradicating *Mycobacterium tuberculosis* bacillus. Along the manuscript, several concepts involved in human tuberculosis are explored in order to understand the big picture, including infection and disease dynamics, animal modeling, liquefaction, inflammation and immunomodulation. Scientists should take into account all these factors in order to answer questions with clinical relevance. Moreover, the inclusion of the concept of a strong inflammatory response being required in order to develop cavitary tuberculosis disease opens a new field for developing new therapeutic and prophylactic tools in which destruction of the bacilli may not necessarily be the final goal.

Along this short review, we would like to explore the gap between medical doctors and basic scientists in terms of their point of view regarding the tuberculosis disease (TBD), to revise several concepts involved in human tuberculosis including the problems of experimental modeling, and to point out why we shall consider including immunomodulation in novel treatment strategies.

## An overview of TB research, a tendentious focus on the bacillus

Since the significant renewal of interest in TB research in the early 1990s, work in this field has tended to focus on the eradication of *Mycobacterium tuberculosis* at different biological scales. Concentrating specifically on the nature of the bacillus, and as a direct result of the development of the whole field of “-*omics*” resources, significant efforts have been expended to identify metabolic pathways that may be susceptible to targeting by drugs and to discern its role in terms of identification by the host's immune system (Brennan, 2003). One of the major victories in this regard has been the identification of the paramount role of ESAT-6 in terms of identification by the immune response, virulence, and its absence in the BCG vaccine (Andersen and Doherty, 2005).

At a cellular scale, the hottest topic has involved discerning how the bacillus is able to subvert both the innate immune response, by manipulating the different mechanisms designed to kill, or at least detect the presence of pathogens; and the adaptive immune response, by delaying its induction or masking detection of the bacilli (Flynn and Chan, 2001; Axelrod et al., 2008). In this regard, a great deal of research has focused on the main actor, namely the alveolar macrophage and, of course, the dendritic cells (Egen et al., 2008; Korbel et al., 2008; Farinacci et al., 2012; Kapina et al., 2013). Neutrophils have proven a less attractive target, although there are always peaks of data highlighting their role (Tan et al., 2006; Martineau et al., 2007; D'Avila et al., 2008), probably because of their low survival times, which makes difficult to detect a relationship with *M. tuberculosis* and it's long doubling time. Another chapter in this field concerns the role of fibroblasts, mainly because of their excellent behavior as “*ex vivo*” systems (Hernandez-Pando et al., 2000; Gonzalez-Avila et al., 2009). Indeed, it appears that fibroblasts could be the hidden link that explains the invisibility of the bacilli during certain phases of an infection. However, this proposal is currently awaiting validation in “*in vivo*” models. A similar situation is found for epithelial or endothelial cells, although very little attention has been paid to the paracrine role of these cells (Coma et al., 2006). In this regard, it is not been clearly established that *M. tuberculosis* is a slow organism (Cox, 2004) and that the “lack of quickness” of the host might be proportional to the slow speed of the pathogen.

The tissue scale has been largely influenced by the presence of hypoxia in the necrotic tissues that surround bacilli in a typical lesion, with an elegant concept coming from the “*in vitro*” acellular experiences of the group of Wayne and the ability of the bacilli to adapt to this stressful environment by becoming dormant or non-replicating (Wayne, 1994). Although this has led to the development of new drugs and “sterilizing” vaccines, this idea has not been well contrasted with the usual oxygen levels at which subcellular structures work (Mochizuki, 1990). The conclusion is that, like many other intracellular pathogens, *M. tuberculosis* always lives under hypoxic conditions, either when inside an isolated cell or when in a granuloma, whereas the high aerobiosis present in laboratory flasks is completely artificial. The concept of persistency is more intriguing as there is a point at which drugs become less and less effective. This seems to be related to some sort of adaptation to the environment mechanism of the bacilli rather than to the acquisition of resistance(Gomez and McKinney, 2004; Dhar and McKinney, 2010). As such, the question arises as to whether this phenomenon could be related to some type of interaction between drugs and tissues, or even the interaction of drugs with the immune system.

In this regard, there is little information at the intersection of what are currently considered to be two distinct worlds: the vaccine and the chemotherapy one. As such, very little effort has been devoted to understanding how the immune response can influence the mechanism of action of chemotherapy, and vice versa.

At the “*in vivo*” scale, one of the main drivers of experimental modeling is the need to obtain a prophylactic or therapeutic strategy able to reduce the bacillary load, even if since now this has not been linked to the avoidance of infection, or at least lead to a minimal peak in bacillary load followed by sterilization of the tissues (Jung et al., 2005).

The concept of virulence can be located at this level. It is widely considered that a virulent strain is one with a high ability to disseminate throughout the population. However, the only way to quantify this is by molecularly characterizing strains isolated from active tuberculosis cases. In contrast, the laboratory concept of virulence, or “fitness”, has always been related to the speed of bacillary growth in an acellular medium (Comas et al., 2012); or in cellular systems (Rey-Jurado et al., 2011) or tissues in the case of “*in vivo*” modeling (Caceres et al., 2012). This latter definition should perhaps be reconsidered.

## The clinical viewpoint

On the other hand, clinicians focus on a very different aspects of human tuberculosis, basically in detecting and treating a case of TBD as soon as possible, as from a medical point of view, there is no risk to life and no risk of transmission in the absence of TBD (Long and Faust, 1941; Rieder et al., 1989).

To be more precise, a clinician's first wish would probably be to have the quickest possible test, with a high specificity and sensitivity for TBD detection, in order to be able to apply it routinely without depending on the semiology of the disease in the subject. In this regard, the paradox is that TBD seems to surpass the clinical challenge of making a good diagnostic orientation—the problem is that big and the symptomatology of TBD that non-specific and confusing—. Indeed, regions with a high incidence, MD students are instructed to consider TB when faced with almost any sort of symptom: “TBD can be anything: TBD must be suspected when facing any clinical case” (Zenner et al., 2013).

On the need of new treatment options, it implies the need for a good geopolitical environment first, which might be a big problem. In terms of the current available treatments, we still might learn a lot and could ameliorate them. Moreover, anybody out from the field could consider the current priority of the WHO, namely the Directed Observed Treatment Strategy (DOTS) (Cox et al., 2008) as “throwing in the towel”. It is not the case. Still what is needed is a clearer and more focused view of the main problem, which is a better distribution and adhesion to the current standard chemotherapy.

In this sense, to reduce the treatment to 15 days only would be ideal. However, how can we do this without having a robust experimental model of TBD? A similar problem is found in diagnostics: how are we going to develop a new and robust test without a good experimental model of evolution towards TBD?

## The problem of modeling experimental TBD

Surprisingly, there is still no robust and validated model of experimental TBD. Moreover, a structured theory of how TBD can be induced is also lacking and yet even more incredible is the absence of an open and dynamic discussion about this subject. Overall, there is as yet no open dialog between the clinical and experimental worlds: apparently, the experimental world seeks the answers to questions that are of little interest to the clinical one.

In the new era of TB research, the most widely used experimental animal model is the one popularized by Ian Orme, at Colorado State University (Orme, 2005). This is a very easy and robust murine model which can be induced by a low dose aerosol, using a Middlebrook device, and is thus highly reproducible. How we can determine whether a mouse has an infection or disease? The only reliable way is to recall an empirical data based on 60 years' experience of using INH alone for the treatment of infection (Comstock, 1999). As this treatment does not cause INH resistance (Balcells et al., 2006), we suppose in this case that the subject has a bacillary load of less than 5 log10, which is the concentration at which spontaneous mutations are induced (Rastogi and David, 1993). Then, this model provides an acute phase in which the bacillary load reaches similar levels to those found in a mild TBD (from 6 to 7 log10), with the chronic phase being reached when the bacillary load is controlled to around 5–6 log10 (Cardona et al., 1999), which is the higher boundary of the infective level.

Thus, in terms of bacillary load, this model includes the progression from infection to active (mild) disease but lacks one vital aspect, namely the induction of a human-like lesion. For this reason, we concluded some time ago that, in contrast to the commonly held belief in the experimental modeling community, mice are “tolerant” to *M. tuberculosis* infection (Cardona, 2006) rather than being resistant (Cardona, 2010) as they can “live with the bacilli” whilst being weakly “provoked” by it. In other words, they exhibit a proportional immune response that is able to control bacillary concentration without generating a marked lesion and, more importantly, with little or no necrosis, depending on the bacillary strain used (Cardona et al., 2003; Gil et al., 2006).

This is the most important handicap of this model as TB lesions in humans are characterized by the generation of a large quantity of necrotic tissue with a very low bacillary load. In this regard, humans tolerate the presence of bacilli very badly, in other words, bacilli are very provocative and trigger an extraordinary and non-proportional inflammatory response in human tissues.

## Experimental modeling TBD: liquefaction is the clue

The tissue destruction in human TBD is so big that the characteristic lesion in adults is the cavity (Canetti, 1955; Grosset, 2003). This means that the necrotic process has been followed by liquefaction and, once eroded, subsequent mass drainage toward the bronchial tree. Traditionally, liquefaction and cavitation are “… nature's more rapid but more hazardous manner of eradicating the disease” (Lurie et al., 1955). Max Lurie devoted all his life to the study of TBD in a model that was able to reproduce both liquefaction and cavitation, thus effectively mimicking what happens in humans. Lurie was able to establish two different colonies of rabbits: resistant and susceptible. The former were able to develop cavitation and heal and had better survival rates than the latter, which did not develop cavitation and had much higher mortality rates. This model also confirmed that cavities preferably develop in the apical lobes by way of experiments which showed that keeping *M. tuberculosis* infected rabbits erect in a harness for 11 h each day produced cavities in the upper lobes rather than in the back of the lungs (Medlar and Sasano, 1936).

Lurie's work was unfortunately truncated after losing both colonies in a disastrous fire. However, Arthur Dannenberg inherited his legacy and extended his experiments, building a structured theory about the evolution from infection to disease (Converse et al., 1996; Dannenberg, 2006). Unfortunately, various mechanistic concepts were difficult to substantiate in this theory as this would require a relatively large investment to develop the tools needed to do so and integrate the model with updated concepts in immunology. The rabbit model of Lurie and Dannenberg has been largely substituted by the mouse model, which helps us to understand the process of infection and immune acquisition against *M. tuberculosis* (Cooper, 2009) but gives no clue as regards the induction of TBD. The reason is that although this model is not able to develop human-like lesions, it has been useful enough to define synergies among drugs and define the length of the treatment required to sterilize the lesions (Lecoeur et al., 1989). Technically speaking, the behavior of the extracellular population, present in the necrotic tissues and growing in the liquefacted tissue, could be determined using “*in vitro*” systems. Indeed, it was Jacques Grosset, probably one of the most experienced TB experimental modelers ever, who highlighted from the very beginning the interest in softening necrotic tissue to allow the entry of oxygen and induce active extracellular growth of the bacilli in the liquefacted product (Grosset, 1980, 2003).

## Liquefaction? cavitation? is everything about dynamicity?

Recent clinical trials have failed to demonstrate the sterilizing activity of moxifloxacin, which would be key for reducing the length of TBD treatment (Jawahar et al., 2013). These clinical trials were conducted on the basis of predictions made using the mouse model (Nuermberger et al., 2004). Because of that, this could be a good opportunity to highlight about the importance to invest in better models developing human-like lesions, especially as regards cavity induction.

The experimental guinea pig model was also been promoted in light of its ability to develop human-like lesions with intragranulomatous necrosis, although it is unable to develop liquefaction and cavitation (McMurray et al., 1996). The extensive work undertaken in macaques, remarcable because its evolutionary proximity to humans, also deserves a mention. Unfortunately, and even if they being so close to humans, macaques have not proven to be robust enough in terms of defining the evolution toward cavitation—the model being unable to make a good prediction of its induction. Pathology evaluation is usually defined after “pathology scoring” (Langermans et al., 2001; Capuano et al., 2003; Verreck et al., 2009), a difficult-to-follow concept that, in the end, reflects an inability to reproduce what finally happens in humans: as no equivalence has been drawn between this pathology scoring and what has been described for more than a century in Pathology Departments.

PET studies have given some degree of dynamicity to the process. The ability to observe the same subject using such a potent imaging technology, allows the same lesion to be monitored over time. This experience has been so stimulating that it has led the authors to enlist different phases in the evolution from infection to disease (Barry et al., 2009). However, this raises the question of the relevance of the concept named “rephasing”. The previous distinction between infection and disease provided definitive support to the clinician when deciding to treat with one drug (in those countries where it is affordable) or three or more drugs in order to avoid the induction of drug resistance. So what is the use of this rephasing? Would it not be more interesting to look at the evolution towards cavitation in experimental models like the one developed in rabbits?

## Experimental modeling: the anatomy and the volume might be crucial

Interestingly enough, other groups of modelers have appeared, producing two new models, using goats and cows (Buddle et al., 2005; Domingo et al., 2009; de Val Perez et al., 2011), in which cavitation can be effectively induced and also the infection can be effectively contained, resembling human-like behavior. The growing problem of TB in cattle has permitted to even use the models letting natural infection happen by leaving ill subjects within the herd to infect healthy ones(Ameni et al., 2010).

Finally, another model using minipigs was developed based on the concept that a big animal should be less tolerant to bacilli than a small one (Cardona, 2006, 2010), as a cavity could be never reproduced in a mouse because it would be larger than the animal's entire body. In this regard, what changes in a large compared to a small mammal is not the size of the cells but the size of their organs, in other words it is simply a question of economy and the ability of large animals to “cut from healthy tissue” to stop the infection progression.

With this aim in mind we started to work with “specific pathogen free” (spf) minipigs in order to avoid any interference from the natural infections that usually occur in even the healthiest animals. To our dismay, these minipigs were unable to induce cavitated lesions, at least in the period of study (up to 23 weeks)(Gil et al., 2010) and small lesions (of the same size as those found in mice, from 0.5 to 2 mm of diameter) were found instead. However, these lesions tended to be encapsulated even being so small, demonstrating the presence of a profibrotic environment in their lungs. This was related to the intralobular septae, a lung structure never been taken into account in the field of TB previously. This structure is only present in the lungs of large mammals (thus being absent in mice, guinea pigs, rabbits or macaques) including humans, with the aim to permit the expansion of such a big organ, and thus helping respiratory function (Fraser, 2005). As for any other collagen-based structure, these septae are very sensitive to mechanical stress and quickly tend to encapsulate any lesion present in the lung, even if only 0.5 mm in diameter.

## TBD and its inflammatory component as the origin of the liquefactive necrosis. more on experimental modeling

**Figure 1:**
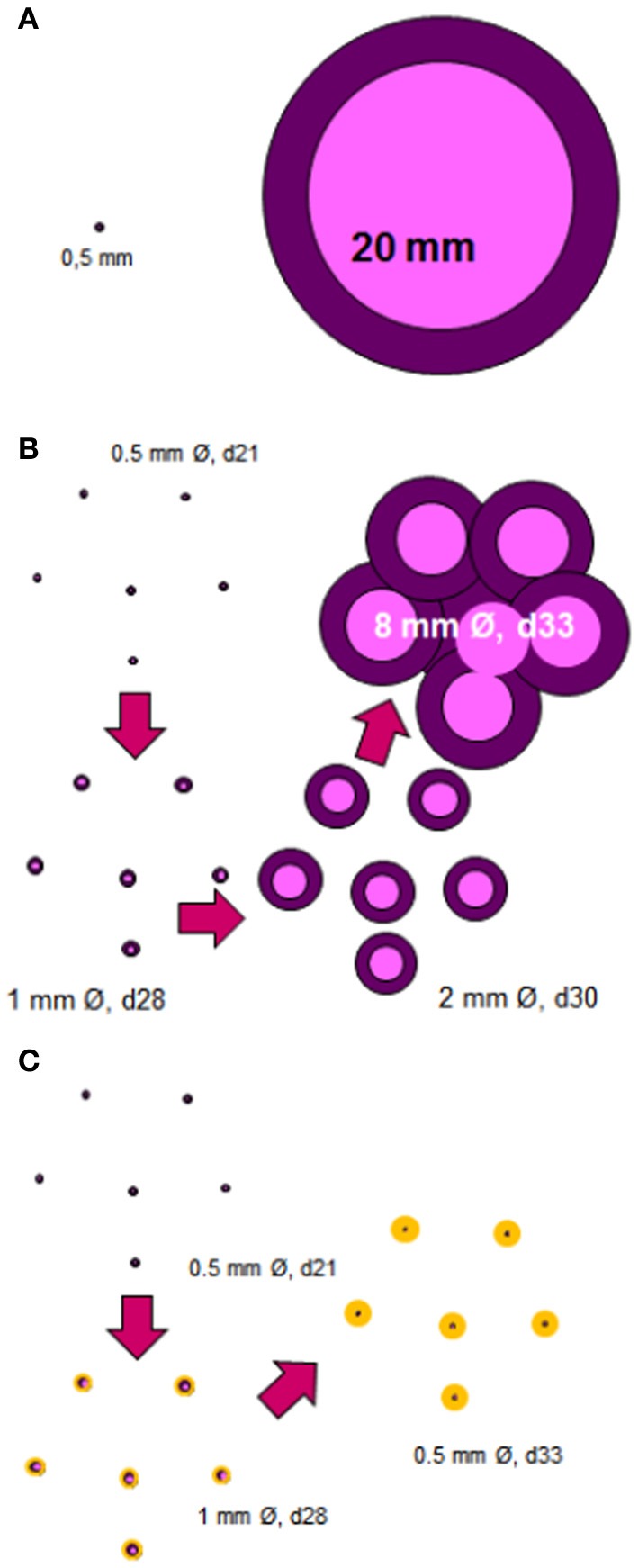
**Induction of cavitated lesions**. The most important question is how a granuloma with a diameter of about 0.5 mm can increase in size to around 25 mm, which is the size required to start a cavity **(A)**. The answer lies in the murine C3HeB/FeJ infection model. In this model, the size of the lesions increases quickly over around 12 days thanks to the massive accumulation of neutrophils, which allows extracellular growth of the bacilli (Marzo et al., 2013), and to the coalescence of some of these lesions **(B)**. The anti-inflammatory preventive intervention should avoid the excessive growth of individual granulomas thus allowing the encapsulation process to occur, and thus avoiding the coalescence and the cavity formation **(C)**.

After many years considering the origin of liquefaction in terms of a fight between profibrotic and antifibrotic mechanisms (Cardona, 2011), we came to realize that the real problem might be mechanical. In light of this, how can the progression of the lesions can be explained? Lesions must evolve from 0.5 to 25 mm, the size at which a lesion has a greater probability of becoming cavitated according to clinical data (Grenville-Mathers, 1947) (Figure 1A).

While studying a mouse model able to develop liquefaction, we identified the data of Kramnik et al. (Yan et al., 2006), who described a model with a similar size of necrosis to the one we experimentally obtained after a short treatment of infected SCID mice (Guirado et al., 2006). At that point we realized that liquefaction was induced in the lesions, but we wanted to reproduce it more robustly as it has seldom been noticed previously in older experiments with Swiss outbred mice (Marzo et al., 2013).

As a result, we started to work with C3HeBe/FeJ mice (those used by the group of Kramnik) and soon realized that a large necrosis was induced in a reproducible manner and that liquefaction occurred as happened in both SCID and Swiss mice. After effectively confirming the reproducibility of the induction of liquefacted lesions, we decided to characterize their evolution. This study showed that individual lesions were able to increase their size exponentially thanks to the massive attraction of neutrophils. The presence of different lesions together meant that large lesions (up to 8 mm in diameter) could form within a week (Marzo et al., 2013) (Figure 1B). What was crucial was the rapid and massive entry of neutrophils into each lesion, showing that liquefaction is a consequence of an excessive inflammatory response, but curiously is not the origin of the extracellular growth of the bacilli; excessive initial neutrophilic infiltration and Neutrophil extracellular traps (NETs) induction supports the initial phases of extracellular bacillary growth (Marzo et al., 2013). Interestingly enough, this phenomenon had already been described by Laennaec, who claimed that both an intense inflammatory response and the coalescence of different lesions were the origin of cavitated lesions, as far back as the 19th century (Hamraoui, 2006).

**Figure 2:**
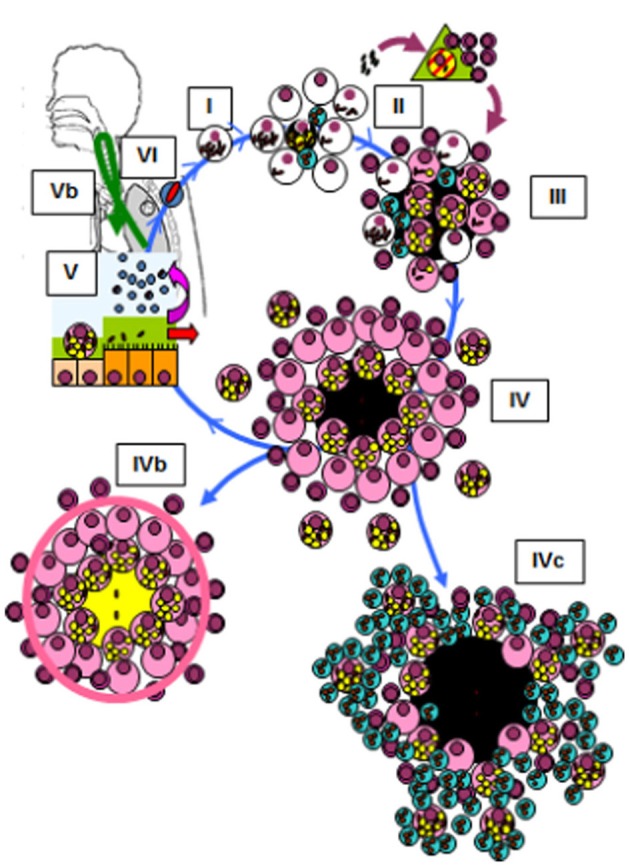
**Life cycle of *Mycobacterium tuberculosis* in the lungs according to the dynamic hypothesis (Cardona, 2009; Cardona and Ivanyi, 2011)**. Modified figure from the original found in Cardona and Ivanyi (2011). Published with the permission of both the author and the editor. (I) After transmission by aerosol, *M. tuberculosis* settles in the alveoli. (II) *M. tuberculosis* grows inside macrophages, causing their necrosis. These cells cause an almost inexistent inflammatory response, and the liberated bacilli are simply phagocytosed by neighboring macrophages, causing no lesion. This phase, which we have named “unicellular” (I), allows the bacilli complete freedom to constantly cause new infectious foci, even in those hosts that have optimal cellular immune responses. This is because, as they do not induce an inflammatory response, these original foci cannot be detected by specific lymphocytes. Once the inflammatory response is sufficiently intense due to the large number of neighboring infected macrophages, cellular traffic between the alveolar space and the capillary allows drainage of the bacilli toward the lymphatic vessels and regional lymph nodes, where antigenic presentation and lymphocytic proliferation take place (II). These lymphocytes are attracted to the inflammatory foci, where they activate the infected macrophages and destroy the bulk of the bacilli (around 90%); the survivors become non-replicating and rest inside activated macrophages or necrotic tissue (III). Once bacillary growth has been controlled, the “cleaning” phase starts. This phase is characterized by phagocytosis of the necrotic debris by the activated macrophages, which retain even more non-replicating bacilli and become “foamy” by accumulating cellular debris (IV). These foamy macrophages are then progressively drained with the alveolar fluid toward the bronchi, where they are destroyed. The bacilli contained therein pass into internal aerosols (V) and are able to reinfect tissue (VI), although they are mainly drained toward the intestinal tract (VI). This cycle can be interrupted by encapsulation, which isolates the granuloma. This process occurs as a result of the intralobar septae, which contain fibroblasts that are very sensitive to the mechanical changes caused by intraparenchymal lesions (IVb). Some hosts can develop an intense neutrophilic response, which is the origin of cavitated lesions (IVc).

The Dynamic Hypothesis of the nature of latent infection (Cardona, 2009) suggests that constant endogenous reinfection is needed for the bacilli to persist in tissue (Figure 2). A reinfection process that takes into account the special milieu at the upper lobes, where the induction of liquefaction and cavitation is possible. In agreement with the epidemiological data, this Hypothesis highlights how the host's drainage system is able to eliminate those non-replicating bacilli that are able to resist both the immune response and chemotherapy, thus making reinfection much less likely with time. The presence of different lesions of a similar age (Figure 1B) might be favored in the upper lobes due to the lower density of the capillary network and the larger size of the alveolar space (Glenny, 2009), both of which favor the local accumulation of lesions (Figure 1B).

## Modeling the role of the different cell-types intervention in the inflammatory response might be a chance for a future therapeutic interventions against TB

As shown in the Figure 2 different cell types interact with *M. tuberculosis*, thus being responsible for the neutrophilic attraction. This aspect has been extensively reviewed recently (Lowe et al., 2012). Briefly, the attraction of the neutrophils at the site of infection is seen at very early stages, attracted by different chemo-attractant cytokines or chemokines synthetized by alveolar macrophages, epithelial cells, γδ T cells or themselves. Th17 cells do so in the immune phase. Interestingly, as recently reviewed, IL-17 acts as an effector molecule similar to IFN-γ after BCG vaccination and *M. tuberculosis* infection, contributing to protection against active TB depending or not on IFN-γ (Li et al., 2012). So far, the role of neutrophils have been fluctuating between its beneficiary role both by directly killing the bacilli or modulating a bactericidal destruction in macrophages; or deleterious by disseminating the infection, being even considered as a “Trojan horse” (Lowe et al., 2012). The direct deleterious role demonstrated by our group has not been considered so far, thus giving a total new view in the field(Marzo et al., 2013).

The concept of modulating the inflammation to achieve a better outcome in TB patients is not new, as is a common practice in daily clinical management of certain forms of TB and under certain conditions (meningitis, pericarditis, TB immune reconstitution inflammatory syndrome)(Lawn et al., 2013). In fact, an extensive review has been published regarding this topic, taking into account the potential use of pro-inflammatory agents, including therapeutic vaccines, immunosuppression agents to reduce inflammation, and effector cytokines to enhance microbicidal effects, all of them in different phases of development. The conclusion is that pro-inflammatory or anti-inflammatory adjuvants can be given, depending on the background of the patient. Overall indicating that an individualized diagnosis and pattern recognition analysis must be done in order to use the best combination (Uhlin et al., 2012).

Taking into account the paramount role of neutrophil infiltration in the evolution of the infection toward disease, our group has used the Kramnik-like experimental model to assay the use of non-steroidal anti-inflammatory drugs (NSAIDs) both in a preventive and therapeutic way, showing a clear beneficial profile in terms of reduction of the lung infiltration, reduction of bacillary load and increase in the survival time (Vilaplana et al., 2013). Considering the wide use and experience in those kind of drugs and the strong security profile, the inclusion of NSAIDs could be easily extended to all TB patients, and especially in those MDR and XDR were the physical progression of the lesions is that important that needs surgical interventions (Ivanyi and Zumla, 2013). In this regard, we do believe that the induction of a balanced immune response is paramount. This is able to avoid an excessive inflammatory response that lead to a self-growing cycle of bacillary growth that potentiates the inflammatory response, which finally induces cavitation.

Finally, these findings also support the concept of some sort of genetic predisposition of the host to develop such an excessive non-proportional inflammatory response (Tobin and Ramakrishnan, 2008) (Figure 2). This could give lead to diagnostic tool that would allow the susceptible population to be identified and, as such, several recent studies have identified remarkable differences between infection and disease in terms of inflammatory response, mainly related to the neutrophil population (Berry et al., 2010).

## Conclusion

After reviewing the current status of experimental modeling in TB and attempting to consider the findings from a clinical viewpoint, the lack of sufficient information concerning the induction of liquefaction as a key mechanism in the induction of TBD becomes clear. Interestingly, a known model in mice (the Kramnik-like model) appoints to a reliable explanation for this process and suggests that the inflammatory response might play an important role on it. More resources should be dedicated to explore these concepts as well to promote more studies including modeling experiments using large mammals to better mimic human tuberculosis.

### Conflict of interest statement

The authors declare that the research was conducted in the absence of any commercial or financial relationships that could be construed as a potential conflict of interest.
